# Comparing Minimally Invasive and Conventional Approaches to DIEP Flap Harvest: A Matched-Pair Analysis from a High-Volume Center

**DOI:** 10.1007/s00266-025-05163-6

**Published:** 2025-08-11

**Authors:** Florian Bucher, Martynas Tamulevicius, Nadjib Dastagir, Moritz Milewski, Louisa Jutta Dietz, Peter Maria Vogt, Khaled Dastagir

**Affiliations:** Department of Plastic, Aesthetic, Hand and Reconstructive Surgery, Hannover Medical School, Carl-Neuberg-Strasse 1, 30625 Hannover, Germany

**Keywords:** Autologous breast reconstruction, Minimally invasive, DIEP flap

## Abstract

**Aims:**

Several approaches aim to reduce donor site morbidity in harvesting autologous deep inferior epigastric perforator (DIEP) flaps. This study aimed to demonstrate a short fasciotomy technique for DIEP flap harvest and to quantify the outcomes, analgesia requirements, and time to mobility compared with the conventional approach.

**Methods:**

This retrospective, single-center study included 22 patients who underwent unilateral breast reconstruction with autologous DIEP flaps between November 1, 2023 and March 1, 2025, performed by a single surgeon. Patients undergoing minimally invasive DIEP flap harvest were matched with those receiving conventional DIEP flap harvest based on key variables and characteristics.

**Results:**

Eleven patients underwent minimally invasive DIEP flap harvest with a mean operating time of 323.54 minutes. The mean length of fasciotomy was 2.79 cm in the minimally invasive group and 9.72 cm in the conventional group. The time to first mobility was 1.18 days in the minimally invasive cohort and 2.09 days in the control group (*p* = 0.0013). The postoperative mean total strong opioid requirement was 18 mg in the minimally invasive cohort and 42 mg in the control group (*p* < 0.001). Patients in the minimally invasive cohort were discharged significantly earlier, with a mean of 5.2 days (*p* < 0.0001). No flap loss occurred in either group.

**Conclusions:**

Minimally invasive DIEP flap harvesting using small fasciotomies is a safe and promising technique that minimizes functional donor site morbidity and offers reliable outcomes, including faster mobilization, reduced postoperative opioid use, and shorter hospital stays.

**Level of Evidence III:**

This journal requires that authors assign a level of evidence to each article. For a full description of these Evidence-Based Medicine ratings, please refer to the Table of Contents or the online Instructions to Authors www.springer.com/00266

## Introduction

Breast cancer remains the second most common cancer worldwide, accounting for 670,000 deaths annually according to the World Health Organization (WHO) [1]. It is the most frequent malignant neoplasia among women globally, with one in eight women at risk of developing invasive breast cancer during their lifetime. Despite improvements in early detection and treatment, breast cancer continues to cause significant mortality [2]. Treatment strategies require a multidisciplinary approach. While breast-conserving surgery is increasingly common, a substantial number of patients still undergo mastectomy, often accompanied by chemotherapy and radiation, depending on local guidelines [3]. Following mastectomy, breast reconstruction is most commonly performed using a direct-to-implant approach. However, in recent years, there has been a noticeable increase in autologous breast reconstruction using deep inferior epigastric perforator (DIEP) flaps [4, 5]. Autologous reconstructions are gaining popularity because they offer superior outcomes in terms of aesthetics, a natural appearance, long-term results, and patient satisfaction compared with other reconstruction options, especially implants [6, 7]. To harvest the DIEP flap pedicle, it is necessary to split the fascia and abdominal wall muscles below the arcuate line. This can lead to abdominal wall weakness due to denervation, increased short-term discomfort and pain, and a higher risk of herniation and bulging [8].

Fascia-sparing surgical techniques reduce donor site morbidity but also result in shorter pedicle length and smaller vessel diameter, making microsurgical anastomosis more challenging. This can limit flap mobility and reduce the margin for surgical error [9]. Recently, laparoscopic and robotic-assisted DIEP flap harvest techniques have been proposed as methods that may offer sufficient pedicle length and diameter while minimizing donor site damage [10]. Laparoscopy can be performed extraperitoneally, but it has notable limitations, including restricted degrees of freedom for vessel preparation, a two-dimensional view, and constrained ergonomics [11]. Robotic flap harvest, on the other hand, is performed transabdominally, extending the surgical approach into the peritoneal cavity and potentially introducing severe complications [12]. Additionally, both laparoscopic and robotic techniques lack tactile feedback, which can further complicate vessel preparation [10].

This study aimed to compare a minimally invasive DIEP flap harvest technique with the conventional approach and to evaluate outcomes, analgesia requirements and time to mobility in a high-volume center in northern Germany.

## Methods

### Study Design

A retrospective descriptive analysis was conducted using the registry of an academic tertiary high-volume center in northern Germany, covering the period from November 1, 2023, to March 2025. Patients included in the study were presented to the local multi-professional interdisciplinary tumor board (ITB) and either required mastectomy for treatment of invasive breast cancer or elected mastectomy based on BRCA1 or BRCA2 gene mutations. Patients who underwent minimally invasive DIEP flap harvest were matched with patients who underwent conventional DIEP flap harvest based on key variables and shared characteristics such as age, body mass index (BMI), comorbidities, and prior radiation or chemotherapy.

All patients or their legal representatives signed the informed consent permitting the use of their anonymous data. Because this is a retrospective analysis of an anonymous database using routine data, approval by the local ethics committee was not required.

### Study Population

Patients included in this retrospective matched-pair analysis were seen during a dedicated consultation hour for breast reconstruction and were presented to the local multi-professional interdisciplinary tumor board. The cohort comprised patients who underwent unilateral breast reconstruction using a unilateral DIEP flap, either as a primary or secondary reconstruction, and were over 18 years of age. To be include in the study, patients must not have had any prior abdominal surgery or preexisting any abdominal pathology before the reconstruction.

The exclusion criteria were an age of less than 18 years, bilateral DIEP flap reconstruction, previous abdominal surgery, untreated abdominal wall pathologies such as hernias, and insufficient documentation.

### Statistical Analysis

Statistical analyses were conducted using GraphPad Prism 10.1.2 (GraphPad Software, Inc., La Jolla, CA, USA), Microsoft Excel (Microsoft Corp., Redmon, WA, USA), IBM SPSS (IBM Corp., Armonk, NY, USA), and Numbers (Apple Inc., Cupertino, CA, USA). Descriptive statistics are presented as numbers (percentages) and medians (interquartile range). Nominal or ordinal variables were compared using Fisher’s exact test. Distribution of metric data was evaluated using Shapiro–Wilk test, and based on the findings, Mann–Whitney test or Student’s *t* test were performed. A *p*-value < 0.05 considered statistically significant.

### Minimally Invasive DIEP Flap Harvest

Patients undergoing breast reconstruction with a DIEP flap underwent preoperative perforator mapping using duplex ultrasound. This dynamic imaging modality enables visualization of the perforator’s course and flow characteristics. The choice of the surgical approach was based on standardized selection criteria: If a single perforator with sufficient flow and short intramuscular course was identified, then minimally invasive DIEP flap harvest was planned. In cases where multiple perforators were needed because of inadequate caliber or blood flow, or if a long intramuscular course was detected, conventional DIEP flap harvest was selected. If necessary, the minimally invasive approach could be converted to the conventional technique at any point during surgery.

All surgeries were performed using a standardized two-team approach led by the senior author, who performed the flap harvesting and microsurgical anastomosis. The DIEP flap was elevated from lateral to medial. After perforator identification, the dominant perforator was followed through a longitudinal incision of the rectus fascia. In the conventional approach, this involved a 10- to 12-cm longitudinal incision of the rectus fascia, followed by trans-muscular dissection using bipolar forceps. By contrast, the minimally invasive approach used only a 3-cm longitudinal incision, followed by trans-muscular, antegrade dissection of the perforator with bipolar forceps. Dissection continued until the vessels branched from the external iliac vessels, ensuring adequate pedicle length and caliber. If necessary, a second vertical incision was made at the lateral margin of the rectus abdominis muscle to lift the muscle using a retractor and continue vessel dissection. The conventional approach typically included one to two perforators, while the minimally invasive approach required a single dominant perforator. The nerve supply to the rectus abdominis muscle was carefully preserved.

The internal mammary vessels at the level of the third intercostal space were used as recipient vessels, which involved partial removal of the fourth rib.

Microsurgical anastomosis was performed using a surgical microscope (Zeiss Pentero 800S). Arterial anastomosis with the internal mammary artery was done end-to-end using non-absorbable 9-0 sutures. The department’s standardized approach includes antegrade and retrograde venous anastomosis of the internal mammary vein using a microvascular coupler device. Flap perfusion was assessed using indocyanine green angiography before and after flap transfer.

**Fig. 1:**
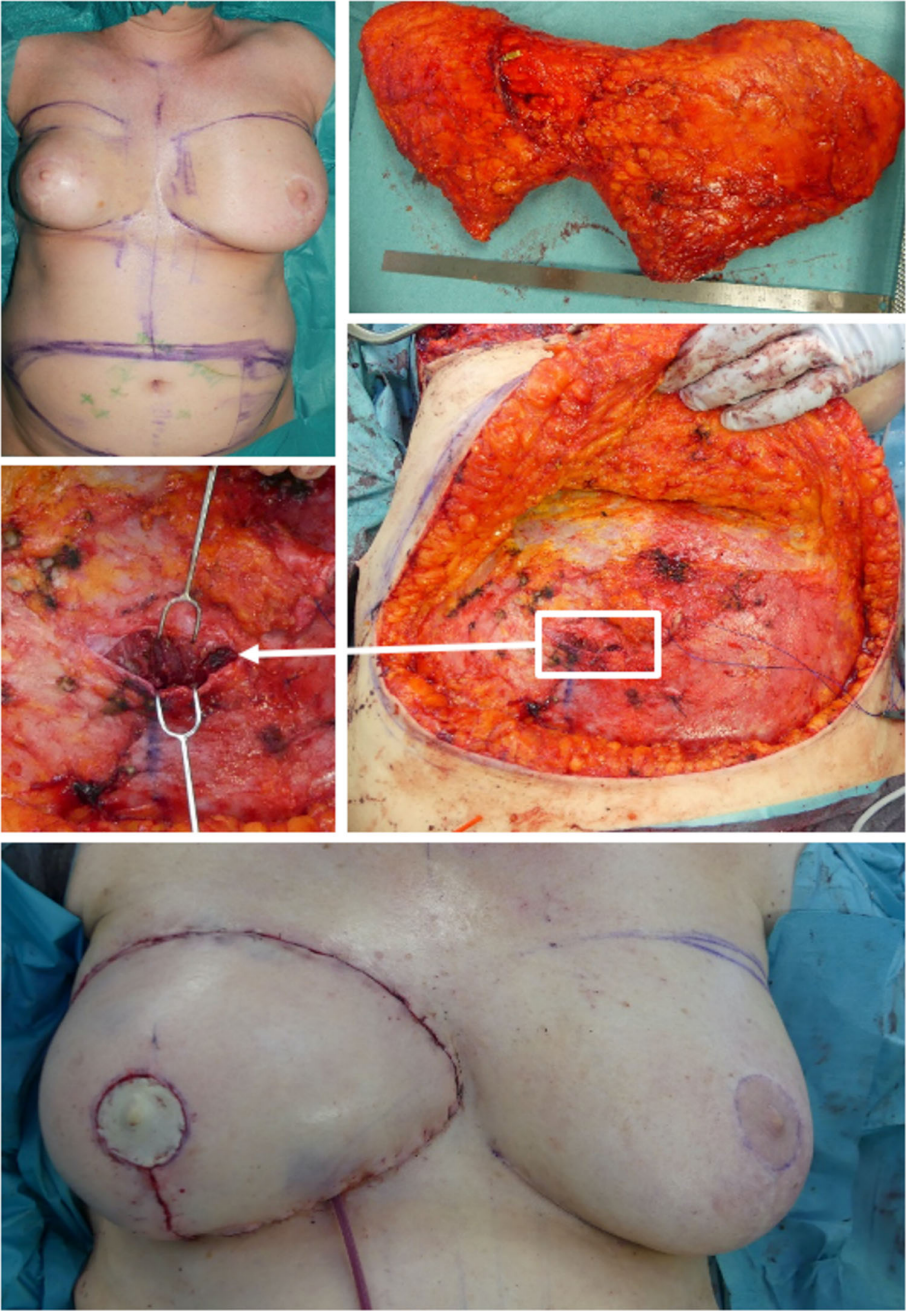
Minimally invasive approach to DIEP flap

**Fig. 2:**
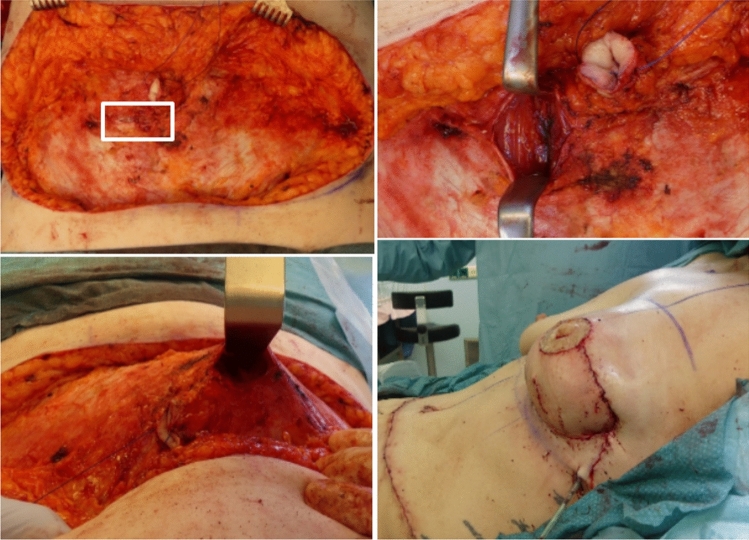
Minimally invasive approach to DIEP flap

Bilayer closure of the rectus fascia was performed in both groups: first using non-absorbable mattress suture (Ethicon Prolene 0), followed by a continuous absorbable running suture (Ethicon PDS 2-0). Drains were placed in both the harvest and donor sites, and skin closure was completed with a continuous intracutaneous suture (Catgut Maricryl 3-0). The surgical technique and results are shown in Figs. 1 and 2.

### Postoperative Protocol

Postoperative perfusion monitoring of the DIEP flap was conducted using near-infrared spectroscopy (NIRS), acoustic Doppler, and clinical assessment of the flap’s skin. Patients received standardized postoperative non-opioid analgesia, corresponding to WHO level 1 analgesia. Analgesic effectiveness was assessed three times per day, and if necessary, opioid analgesia (WHO level 3) was added to ensure adequate pain control. Anticoagulation was managed with low-molecular-weight heparin, and acetylsalicylic acid was administered as an antithrombotic agent for 10 days postoperatively.

## Results

In total, 22 patients were included in this study, equally divided between the minimally invasive and conventional DIEP flap harvest cohorts. The overall mean age was 52.14 years (SD 5.39 years; 41–62 years), and the mean BMI was 24.1 kg/m^2^ (SD 3.21 kg/m^2^; 19.5–29.7 kg/m^2^). There were no statistically significant differences between the cohorts in terms of cardiovascular, renal, endocrine, or psychological disorders. In each group, seven patients underwent prophylactic mastectomy because of a BRCA1 or BRCA2 gene mutation, while four patients underwent mastectomy following a diagnosis of invasive breast cancer. A total of 12 patients received both radiotherapy and chemotherapy. The main comorbidities, namely cardiovascular diseases, diabetes mellitus, endocrine disorders, immunological disorders, and psychological disorders, showed no statistically significant differences between the two groups. The patients’ main characteristics and comorbidities are summarized in Table 1.

**Table 1:** Patient characteristics

	Conventional	Minimally invasive	Significance	*p* value
*Patient characteristics*				
Age (years)	52.5 (44–58)	51.7 (39–62)	No	0.3210
BMI (kg/m^2^)	23.9 (21.2–29.8)	24.3 (23.3–30.1)	No	0.1364
Smoking history	9 (18.2%)	1 (9.1%)	No	0.5568
*Pathology*				
BRCA mutation	7 (63.6%)	7 (63.6%)	No	> 0.9999
Disseminated cancer	0 (0%)	0 (0%)	No	> 0.9999
Previous radiation	5 (45.5%)	7 (63.6%)	No	0.4161
Previous chemotherapy	7 (63.6%)	5 (45.5%)	No	0.0785
*Comorbidities*				
Diabetes mellitus	1 (9.1%)	2 (18.2%)	No	0.5568
Cardiovascular disease	4 (36.4%)	2 (18.2%)	No	0.3622
Renal disorder	1 (9.1%)	0 (0%)	No	0.3292
Endocrine disorder	0 (0%)	1 (9.1%)	No	0.3292
Immunological disorder	0 (0%)	1 (9.1%)	No	0.3292
Psychological disorder	1 (9.1%)	2 (18.2%)	No	0.5568

**Fig. 3:**
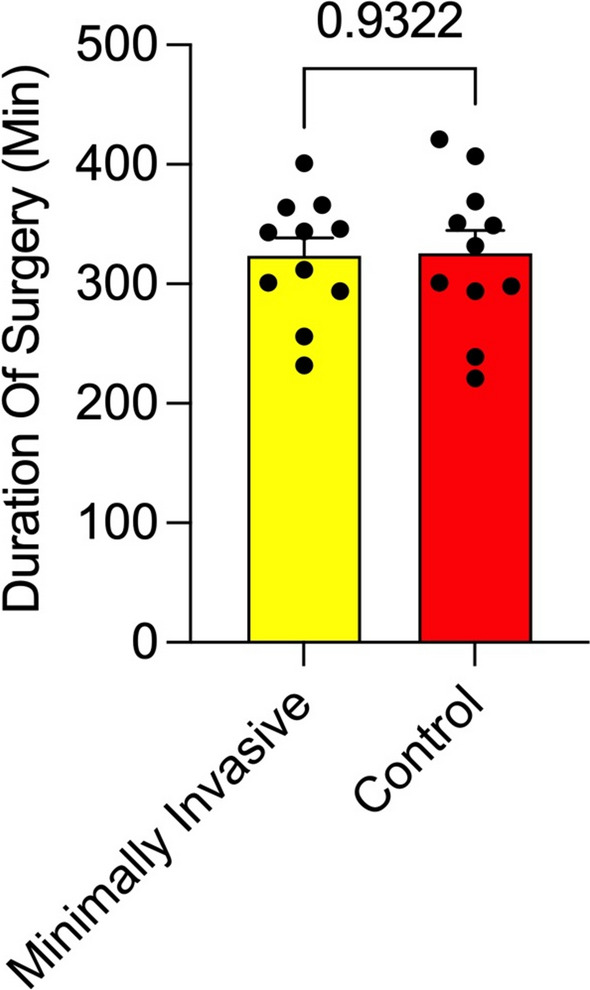
Duration of surgery

The mean operation time was slightly shorter in the minimally invasive cohort, with a mean of 323.54 min (SD 50,2 min; 294–401 min) compared with 325.63 min (SD 62.97 min; 221–421 min) in the conventional group, although this difference was not statically significant difference (*p* = 0.9322) (Fig. 3). The mean length of fasciotomy was 2.79 cm (SD 0.08 cm; 2.7–2.9 cm) in the minimally invasive cohort versus 9.72 cm (SD 0.76 cm; 8–10.2 cm) in the conventional group. Four patients in the minimally invasive cohort required secondary incisions to facilitate flap harvest with a mean length of 3.12 cm (SD 0.17 cm; 2.9–3.3 cm). The mean pedicle length in the minimally invasive flap harvest cohort was 12.18 cm compared to the conventional approach, with 13.14 cm, and did not show statistically significant differences.

**Fig. 4:**
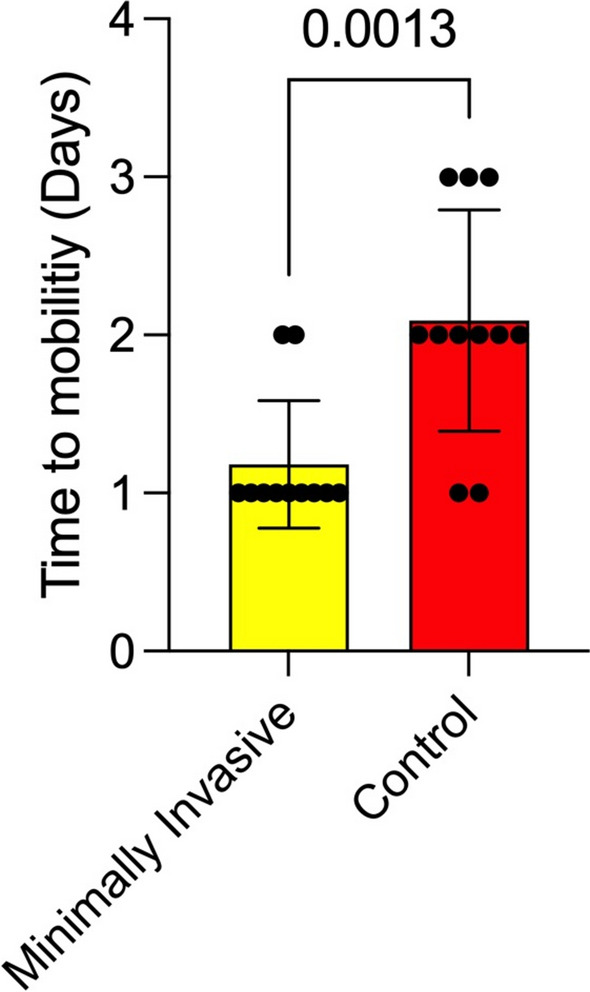
Time to mobility

**Fig. 5:**
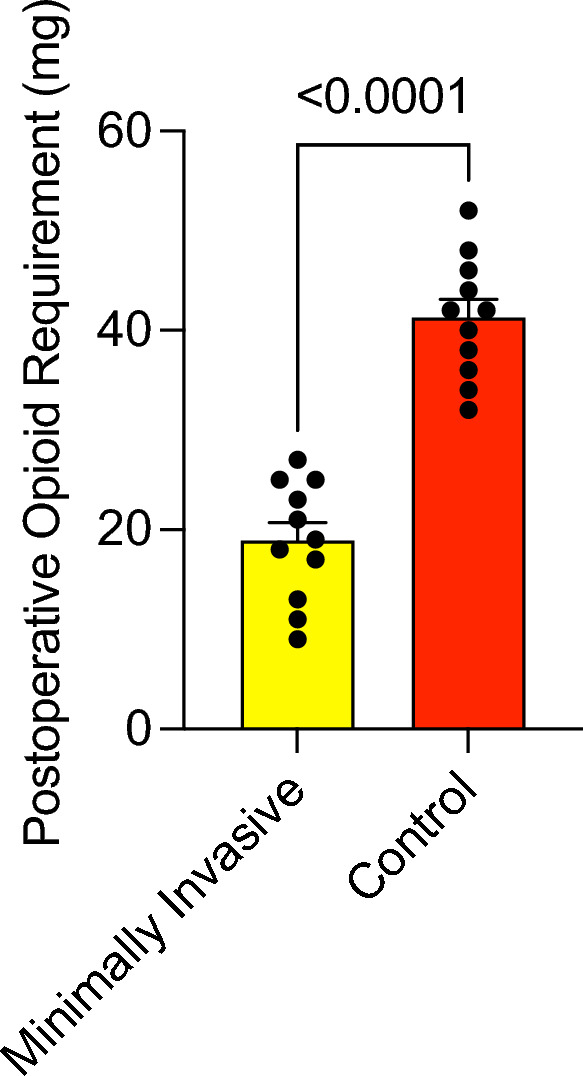
Postoperative opioid requirement (mg)

**Fig. 6:**
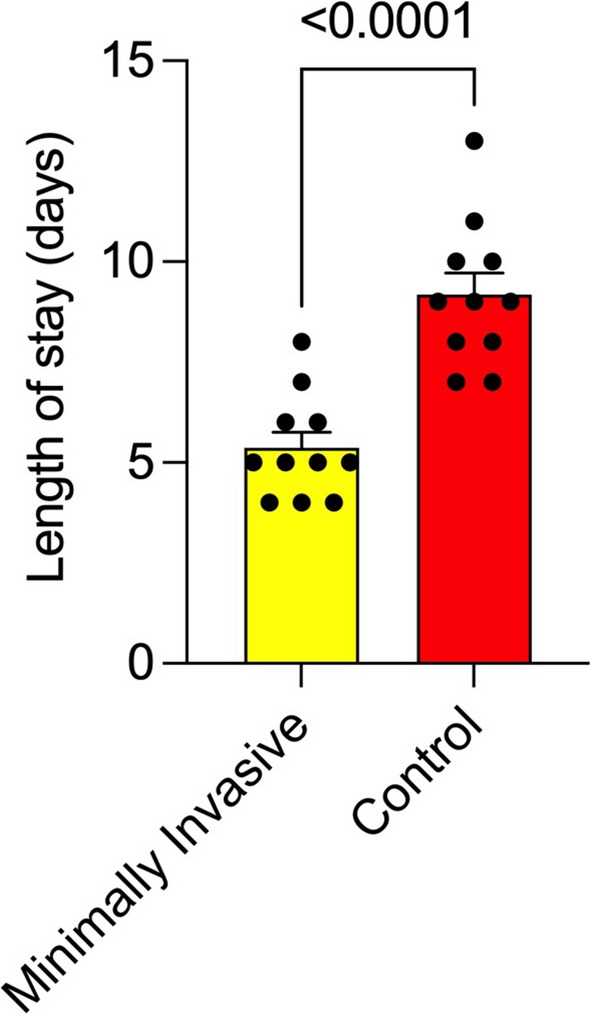
Length of hospital stay

Patients in the minimally invasive group were mobilized significantly faster (*p* = 0.0013), with a mean time to full mobility of 1.18 days (SD 0.4 days; 1–2 days), compared to 2.09 days (SD 0.7 days; 1–3 days) in the conventional group (Fig. 4). The total dose of strong opioid analgesia required was significantly lower in the minimally invasive group with a mean of 18 mg hydromorphone (SD 6.0 mg; 9–27 mg), compared with 42 mg (6.08 mg; 34–52 mg ) in the conventional cohort (*p* < 0.001) (Fig. 5). Additionally, patients undergoing minimally invasive DIEP flap harvesting were discharged significantly earlier, with a mean hospital stay of 5.2 days (SD 1.2 days; 4–8 days), compared with a mean of 9.1 days (SD 1.77 days; 7–13 days) in the conventional group (*p* < 0.0001) (Fig. 6). The characteristics of each surgical approach and its postoperative course are summarized in Table 2. During the 12-month follow-up, no flaps were lost, and no operative revisions were required. Immediate postoperative bulging or herniation due to abdominal wall weakness did not occur in either group. The postoperative complications are summarized in Table 2.

**Table 2:** Postoperative course and complications

	Conventional	Minimally invasive	Significance	*p* value
*Surgery*				
Operating time (min)	325 (221–407)	323 (301–401)	No	0.9322
Length of fasciotomy (cm)	9.72 (8–10.2)	2.79 (27.9–2.9)	**Yes**	**< 0.0001**
Pedicle length (cm)	13.14 (11–16)	12.08 (11–14)	No	0.1522
*Postoperative*				
Total opiod dosage (mg)	42 (34–52)	18 (9–27)	**Yes**	**< 0.001**
Time to mobility (days)	2.09 (1–3)	1. 18 (1–2)	**Yes**	**0.0013**
Length of stay (days)	9.1 (7–3)	5.2 (4–8)	**Yes**	**< 0.001**
*Complications*				
Revision	0 (0%)	0 (0%)	No	> 0.9999
Flap loss	0 (0%)	0 (0%)	No	> 0.9999
Fat partial necrosis	1 (9.1%)	1 (9.1%)	No	> 0.9999
Hematoma	0 (0%)	0 (0%)	No	> 0.9999
Delayed wound healing dor	2 (18.2%)	2 (18.2%)	No	> 0.9999
Abdominal bulging	0 (0%)	0 (0%)	No	> 0.9999
Seroma donor site	3 (27.3%)	2 (18.2%)	No	0.6309

## Discussion

Breast reconstruction using the DIEP flap is the current gold standard for autologous breast reconstruction, offering an aesthetically pleasing restoration of breast shape with a natural tissue appearance [13]. However, donor site morbidity remains a critical factor influencing patient satisfaction. As a result, there has been growing interest in refining the conventional DIEP flap harvest technique to better preserve the abdominal wall by minimizing operative trauma [14, 15].

This study aimed to compare minimally invasive DIEP flap harvest, with a mean fasciotomy length of 2.79 cm, to the conventional DIEP flap harvest technique. A comparable approach was described by Kim et al. [16], although with a larger mean fasciotomy of 6.6 cm. Abdominal bulging is a known complication attributed to abdominal wall trauma during dissection of the DIEP flap perforator, occurring in 3% to 6% of patients as reported in the literature [17, 18]. Weakness of the abdominal wall can result in significant morbidity, including the need for higher and stronger doses of pain medication, particularly opioids, limited mobilization, and the potential for bowel herniation. The surgical technique presented in this study seeks to reduce these complications by performing the DIEP flap harvest with a minimal fasciotomy of less than 3 cm. Notably, no patients in either cohort developed postoperative abdominal wall bulging or herniation during the 12-month follow-up period. As previously demonstrated in the literature, the length and location of the abdominal wall incision are directly associated with postoperative pain intensity and delay bowel movement [19, 20]. However, four patients in the minimally invasive DIEP flap cohort required secondary incisions to facilitate flap harvest.

In this study, patients who underwent minimally invasive DIEP flap harvest required nearly 50% less strong opioid medication postoperatively compared with the conventional control group. As described in the meta-analysis by Rivas et al. [21], mobilization is critical for postoperative recovery following abdominal surgery; however, patients typically spend only a fraction each day mobilized, despite its importance for healing. Postoperative pain is closely linked to mobilization time, highlighting the importance of pain reduction as key role during both surgery and postoperative care [21]. Previous studies have shown that unilateral abdomen-based breast reconstruction reduces postoperative pain and complications [22, 23]. These findings align with our experience, and autologous breast reconstruction using DIEP flaps is routinely performed unilaterally as a standard procedure. The contralateral side may be addressed after a 3-month recovery period. Patients who underwent minimally invasive DIEP harvest reported lower postoperative pain and were therefore able mobilize significantly earlier on day 1, while the conventional group required approximately twice as long. Additionally, patients in the minimally invasive cohort were discharged significantly earlier, with a mean duration of stay of 5.2 days, compared to 9.1 days for the control group. These outcomes are consistent with the current literature. As described by Tazreean et al. [24], early mobilization reduces postoperative complications, accelerates recovery, shortens hospital stay, and improves patient-reported outcomes. Patients undergoing autologous breast reconstruction with DIEP flaps are typically middle-aged women who maintain a high level of activity and place a high value on aesthetic outcomes. The surgical technique described for minimally invasive DIEP flap harvest meets these standards by reducing surgical trauma to the abdomen, thereby minimizing postoperative morbidity. The matched-pair analysis demonstrated the safety of the minimally invasive DIEP flap harvest, showing comparable mean operation times and no significant differences in postoperative complications. The mean pedicle length did not show statistically significant differences between the cohorts. The mean pedicle length for the minimally invasive DIEP flap harvest was 12.18 cm, providing sufficient length for microsurgical anastomosis. No patients required operative revision or experienced flap loss. Similar findings regarding the safety of short fasciotomy DIEP flap harvest were reported by Kim et al. [16]. While Kim et al. [16] utilized both single and dual perforator flaps, the technique described here relies exclusively on single perforator flaps. The choice between single and multiple perforator DIEP flaps depends on the surgeon’s preference and the patient’s anatomy. According to the literature, overall complication rates do not significantly differ between single and multiple perforator DIEP flaps [25]. To ensure optimal flap perfusion, the department’s standard protocol includes indocyanine green angiography (ICG-A) before and after flap transfer. If necessary, an additional perforator may be included, converting the minimally invasive approach into to the conventional technique. As shown in the literature, ICG-A is a reliable and accurate method for intraoperative evaluation of flap perfusion [26].

Recently, laparoscopic and robotic-assisted DIEP flap harvest techniques have been proposed as modern approaches aimed at reducing donor site morbidity [10]. However, both methods lack tactile feedback, offer limited degrees of freedom for vessel preparation, and present ergonomic challenges [11]. Flap harvest may be performed either extraperitoneally or via a transabdominal preperitoneal route, effectively converting the operation into a dual-cavity procedure with the potential for peritoneal cavity complications. The approaches are also associated with longer operative times and increased costs [27]. The use of trocars in these systems can result in abdominal wall injuries, weakening of the abdominal wall, and lead to herniation rates of up to 4.7%. Additionally, there is a risk of iatrogenic intraabdominal organ injury and postoperative peritoneal adhesions up to 12% of cases [28–30]. By contrast, the minimally invasive DIEP harvest technique described in this study does not prolong operative time or increase costs, whereas overall surgical expenses for robotic systems may rise by as much as 25% [31]. The described DIEP flap harvest technique should be considered an incremental improvement of the conventional DIEP flap harvest technique, which has been established over the past decades.

Although the presented results support the feasibility and benefits of the minimally invasive DIEP flap harvest, several limitations must be acknowledged. The retrospective nature of this study inherently limits the strength of the conclusions due to potential selection bias, missing data, and limited control over confounding variables. Also, the relatively small sample size of 22 patients may limit the generalizability of the reported findings. All operations were performed by the senior author, which may introduce single-operator bias and reflect a learning curve influenced by experience with the conventional approach. Additionally, the BMI of the study cohort was lower than that of the general population. Previous studies have identified BMI as a risk factor for postoperative complications, which may therefore be underrepresented in this cohort [32]. This study lacks long-term follow-up including objective and functional assessments such as abdominal wall strength. Lastly, the described technique is limited to single perforator DIEP flap harvests due to minimal fasciotomy length; however, it can be converted to the conventional approach at any point during surgery if necessary. Future studies with larger sample sizes, ideally conducted in a multi-center setting, are needed to provide more robust evidence.

## Conclusion

Minimally invasive DIEP flap harvesting using small fasciotomies is a safe and effective alternative to conventional DIEP flap harvest or the use of robotic and laparoscopic techniques. Patients in the minimally invasive cohort required significantly less strong opioid medication, were mobilized faster, and had a shorter hospital stay. At the same time, the mean operative time and overall complication rates did not differ between the groups.

This approach is best suited for single dominant perforator flaps with antegrade perfusion and a minimally intramuscular course.
